# Supplementary material for: Comparison between Self-Completed and Interviewer-Administered 24-Hour Dietary Recalls in Cancer Survivors: Sampling Bias and Differential Reporting
Source: Nutrients. 2022 Dec 8;14(24):5236. doi: 10.3390/nu14245236 (PMC9781276; doi:10.3390/nu14245236)
Supplement: Supplementary file 1 [file nutrients-14-05236-s001.zip › nutrients-2051005-supplementary.pdf]

Title: Comparison between Self-Completed and Interviewer-Administered 24-Hour Dietary Recalls in Cancer Survivors: Sampling Bias and Differential Reporting

Corresponding author: Rana E Conway

Online Supplementary material

Supplementary Table S1. Logistic regression analyses for method of completing 24-HR (interviewer-completed 24-HR as target group)

Demographic and health characteristics, and use of technology		**Unadjusted**			**Adjusted[a] (n1019)**		
	n	OR	95% CI	P	OR	95% CI	P
Age (years)	1221	1.08	1.07-1.10	**<0.001**	1.09	1.07-1.11	**<0.001**
Gender	1224						
Male		1.00	-	**-**	1.00	-	-
Female		0.65	0.51-0.83	**<0.001**	1.26	0.90-1.76	0.174
Ethnicity	1219						
White		1.00	-	-	1.00	-	-
Non-White		1.40	0.89-2.20	0.149	2.67	1.44-4.94	**0.002**
Highest Level of Education	1145						
Degree or higher		1.00	-	-	1.00	-	**-**
A-levels		1.6	1.03-2.38	**0.036**	2.11	1.29-3.44	**0.003**
GCSE/Vocational		1.8	1.33-2.55	**<0.001**	2.36	1.60-3.46	**<0.001**
No formal qualifications		6.9	4.74-10.02	**<0.001**	5.13	3.31-7.96	**<0.001**
Marital status	1223						
Married		1.00	-	-	1.00	-	-
		1.92	1.44-2.56	**<0.001**	2.69	0.91-2.08	0.137
Divorced/Separated/Widowed		1.02	0.66-1.58	0.945	1.37	0.91-3.00	0.101
Single							
Employment	1217						
Employed		1.00	-		1.00	-	
Retired		2.91	2.21-3.83	**<0.001**	0.94	0.62-1.41	0.771
Other		1.74	1.09-2.81	**0.021**	0.338	0.73-2.42	0.338
IMD (decile)	1161	0.96	0.91-1.00	0.064	0.957	0.898-1.02	0.179
BMI	1160						
Underweight/healthy		1.00	-	-	1.00	-	-
weight		0.95	0.82-1.25	0.710	0.73	0.52-1.03	0.072
Overweight		1.24	0.89-1.74	0.199	1.01	0.67-1.51	0.969
Obesity							

Abbreviations: CI, confidence interval; OR, odds ratio
[a]adjusted for Age, Gender, Ethnicity, Highest level of Education, Marital status, Employment status, IMD, BMI category
